# (−)-Epicatechin-3-*O*-β-d-allopyranoside from *Davallia formosana*, Prevents Diabetes and Hyperlipidemia by Regulation of Glucose Transporter 4 and AMP-Activated Protein Kinase Phosphorylation in High-Fat-Fed Mice

**DOI:** 10.3390/ijms161024983

**Published:** 2015-10-20

**Authors:** Chun-Ching Shih, Jin-Bin Wu, Jia-Ying Jian, Cheng-Hsiu Lin, Hui-Ya Ho

**Affiliations:** 1 Graduate Institute of Pharmaceutical Science and Technology, College of Health Science, Central Taiwan University of Science and Technology, Taichung City 40601, Taiwan; E-Mail: ccshih@ctust.edu.tw; 2 Graduate Institute of Pharmaceutical Chemistry, China Medical University, Taichung City 40402, Taiwan; E-Mail: jwu@mail.cmu.edu.tw; 3 Department of Internal Medicine, Fong-Yuan Hospital, Department of Health, Executive Yuan, Fong-Yuan District, Taichung City 42055, Taiwan; E-Mail: keny71@pchome.com.tw; 4 Jen Li Biotech Company Ltd., Taiping District, Taichung City 41143, Taiwan; E-Mail: jlbioaya@gmail.com

**Keywords:** *Davallia formosana*, diabetes, hypertriglyceridemia, AMP-activated protein kinase phosphorylation, glucose transporter 4

## Abstract

The purpose of this experiment was to determine the antidiabetic and lipid-lowering effects of (−)-epicatechin-3-*O*-β-d*-*allopyranoside (BB) from the roots and stems of *Davallia formosana* in mice. Animal treatment was induced by high-fat diet (HFD) or low-fat diet (control diet, CD). After eight weeks of HFD or CD exposure, the HFD mice were treating with BB or rosiglitazone (Rosi) or fenofibrate (Feno) or water through gavage for another four weeks. However, at 12 weeks, the HFD-fed group had enhanced blood levels of glucose, triglyceride (TG), and insulin. BB treatment significantly decreased blood glucose, TG, and insulin levels. Moreover, visceral fat weights were enhanced in HFD-fed mice, accompanied by increased blood leptin concentrations and decreased adiponectin levels, which were reversed by treatment with BB. Muscular membrane protein levels of glucose transporter 4 (GLUT4) were reduced in HFD-fed mice and significantly enhanced upon administration of BB, Rosi, and Feno. Moreover, BB treatment markedly increased hepatic and skeletal muscular expression levels of phosphorylation of AMP-activated (adenosine monophosphate) protein kinase (phospho-AMPK). BB also decreased hepatic mRNA levels of phosphenolpyruvate carboxykinase (PEPCK), which are associated with a decrease in hepatic glucose production. BB-exerted hypotriglyceridemic activity may be partly associated with increased mRNA levels of peroxisome proliferator activated receptor α (PPARα), and with reduced hepatic glycerol-3-phosphate acyltransferase (GPAT) mRNA levels in the liver, which decreased triacylglycerol synthesis. Nevertheless, we demonstrated BB was a useful approach for the management of type 2 diabetes and dyslipidemia in this animal model.

## 1. Introduction

It is estimated that the population of diabetes mellitus will reach 300 million by 2025. Type 2 diabetes mellitus causes high glucose levels in the blood due to enhanced liver glucose production, or “insulin resistance” (insensitive response to insulin by peripheral tissues). Type 2 diabetes mellitus represents over 90% of diabetes cases [1]. Type 2 diabetes mellitus is involved in numerous factors (such as obesity and family history) [2]. Both genetics and lifestyle play significant roles [3]. Mice were induced with a high-fat diet (HFD) to cause aberrant muscular glucose uptake faults, insulin resistance, hyperglycemia, hyperlipidemia, hyperinsulinemia, hyperleptinemia, obesity, and excess circulating free fatty acid [4].

*Davallia formosana* Hayata (Davalliaceae) in Taiwan’s herbal market is also known as Gu-Sui-Bu, which is substituted for *Drynaria fortune* and is employed to treat rheumatoid arthritis. The former name of *Davalli formosana* in the flora of Taiwan is *Davallia orientalis* C. Chr. (1932–1975) and *Davallia divaricata* Bl. (1975–1991). The official nomenclature has been *Davallia formosana* since 1994 [5]. The bioactive components of *Davalli divaricata* are demonstrated to be davallic acid [6] and flavan-3-ol and proanthocyanidin allosides [7].

**Figure 1:**
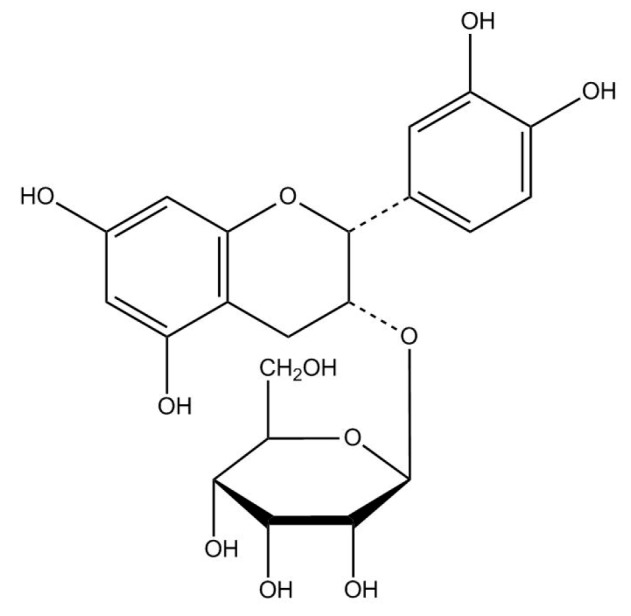
Structure of (−)-epicatechin-3-*O*-β-d-allopyranoside (BB).

It was observed that some of the antidiabetic agents have been shown to be favorable to osteogenesis and decreasing adipogenesis. Metformin has favorable effects on osteoblast differentiation and calcium accretion in streptozotocin-induced diabetes mellitus (DM) rats [8,9]. Moreover, extract of cinnamon bark displayed facilitated bone formation [9,10], which is associated with regulating serum insulin and adiponectin levels and improving insulin sensitivity, thus exhibiting both antihyperglycemic and antihyperlipidemic action. The effects of (−)-epicatechin-3-*O*-β-d-allopyranoside (BB) (Figure 1) from *Davallia formosana* displayed a favorable effect on bone loss; nevertheless, the effects of BB on antihyperglycemic and antihyperlipidemic action remain unknown. Therefore, we investigate the antidiabetic and lipid-lowering effects of BB in mice.

The glucose transporter 4 (GLUT4) plays a vital role in controlling whole body glucose homeostasis [11]. Insulin is secreted after a meal (included carbohydrates), and followed by stimulating glucose transport. In response to insulin and other stimuli, GLUT4 has been shown from intracellular disposition, acutely redistributing to the plasma membrane [11,12]. The upregulation of GLUT4 expression in skeletal muscle was observed in response to exercise in a mouse model to affect glucose levels in blood [11,13]. Thus, treatments that target enhancing GLUT4 proteins are vital points for the reduction of diabetes. Regarding the enhanced movement of GLUT4 from cytosol to membrane, there are some important pathways: (1) the insulin signaling pathway, and (2) the contraction- [14,15] or hypoxia-mediated stimulation of AMP-activated protein kinase (AMPK) [15,16].

AMPK plays a key role in glucose and lipid metabolism. Metformin is an agent in the management of type 2 diabetes, and acts to improve blood glucose control mainly by the inhibition of liver glucose production and increased peripheral glucose uptake [15,17]. Therefore, AMPK activation could have favorable effects in peripheral tissues in type 2 diabetes [15,17].

Thiazolidinediones (TZDs) have been used to treat type 2 diabetes, and belong to peroxisome proliferator-activated receptor γ (PPARγ) agonists. TZDs, including rosiglitazone, decrease glucose levels in the blood [18]. Rosiglitazone acts to enhance insulin sensitivity and protein levels of GLUT4 [19,20]. Fenofibrate (belongs to a PPARα activator) has been used to treat human hyperlipidemia and lower blood triglyceride levels [21,22]. PPARα is a major regulator of genes involved in lipid metabolism [23]. By regulating fatty acid oxidation and lipogenesis, the PPARα agonist leads to decreasing blood triglyceride levels [24]. TZDs and metformin both cause AMPK activation. The difference between TZDs and metformin is that TZDs could enhance adiponectin and cause AMPK activation [25,26]. The enhanced adiponectin levels are involved in reduced hepatic fat pads and ameliorated insulin resistance [26]. Therefore, we employ Rosi and Feno as positive control.

The present study employs C57BL/6J mice on a high-fat diet (HFD) to examine the glucose- and lipid-lowering activities of BB compared with rosiglitazone (Rosi) and fenofibrate. AMPK activity depends on phosphorylation of Thr 172 of α subunits [27]. This study is to monitor the hypothesis that BB could improve expression levels of phospho-AMPK, protein levels of GLUT4, and target gene mRNA levels in diabetes and hyperlipidemia in peripheral tissues following four weeks of administration of BB. Our results imply that enhanced expression levels of phospho-AMPK and protein levels of GLUT4 are key targets of BB to ameliorate diabetic and dyslipidemic events.

## 2. Results

### 2.1. Body Weight, Weight Gain, Diet Consumption, and Weights of Tissue

**Figure 2:**
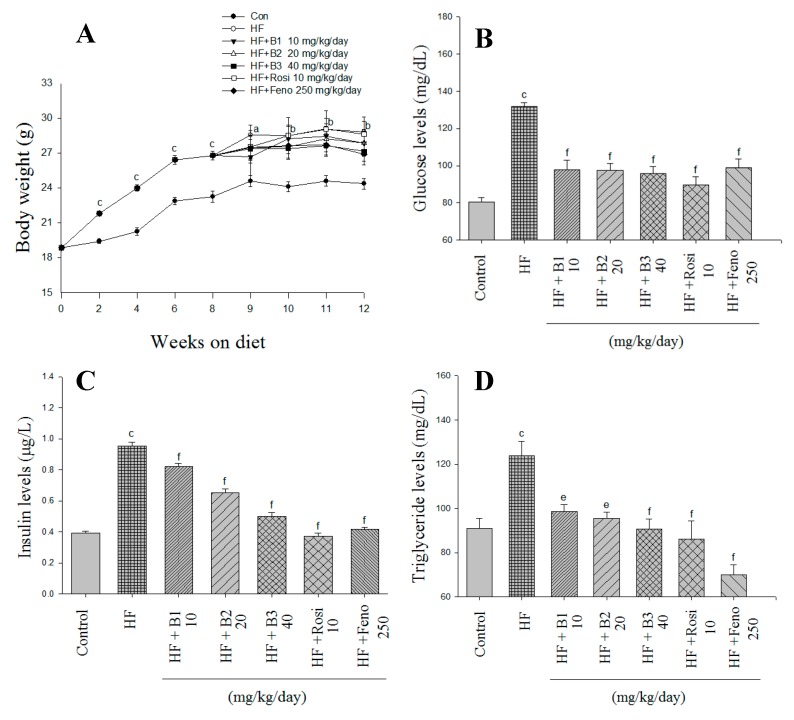
Effects of (−)-epicatechin-3-*O*-β-d-allopyranoside (BB) on (**A**) body weight; (**B**) blood glucose levels; (**C**) insulin levels; and (**D**) triglyceride levels at week 12. Mice were fed with a 45% high-fat diet (HF; high-fat diet control) or low-fat diet (CON) for 12 weeks. After eight weeks of induction, the HF mice were treated with vehicle, or (−)-epicatechin-3-*O*-β-d-allopyranoside (BB), or rosiglitazone (Rosi), or fenofibrate (Feno) accompanied with a HF diet for four weeks. All values are means ± SE (*n* = 9). ^a^ *p* < 0.05, ^b^ *p* < 0.01, ^c^ *p* < 0.001 compared with the control (CON) group; ^e^ *p* < 0.01, and ^f^ *p* < 0.001 compared with the high-fat plus vehicle (distilled water) (HF; high-fat diet control) group. (−)-epicatechin-3-*O*-β-d-allopyranoside (BB): B1: 10, B2: 20, B3: 40 mg/kg body wt; Rosi: rosiglitazone (10 mg/kg body wt); Feno: fenofibrate (250 mg/kg body wt). BAT, brown adipose tissue; RWAT, retroperioneal white adipose tissue; MWAT, mesenteric white adipose tissue; FFA, plasm free fatty acid; visceral fat represented epididymal WAT plus retroperitoneal WAT.

At the beginning, the mean body weights of each group are similar (18.86 ± 0.12 g). After 12 weeks on HFD, mice elicit increased body weight and body weight gain (Figure 2A and Table 1). No difference was observed in body weight when a comparison was made between the B1-, B2-, B3-, Rosi-, and Feno-administered groups and the HF (high-fat control) group (Figure 2A). B3- and Feno-treated mice displayed reduced weight gain compared with HF littermates (Table 1). HF mice consume a HFD (g) much less than CON (control) mice (*p* < 0.001) (Table 1). Food intake of BB-, Rosi-, and Feno-treated groups is similar to that of the HF group. Mice on HFD display increased epididymal white adipose tissue (EWAT) and retroperitoneal WAT (RWAT), visceral fat, mesenteric WAT (MWAT), skeletal muscle, and brown adipose tissue (BAT) weights compared to CON mice (Table 1). All of the BB-, Rosi-, and Feno-treated groups showed reduced EAWT and RWAT and visceral fat weights. B3-treated mice exhibit increases in skeletal muscle weights (*p* < 0.05) while Feno-treated mice display increased liver weights (*p* < 0.001) (Table 1).

**Table 1:** Effects of (−)-epicatechin-3-*O*-β-d-allopyranoside (BB) on absolute tissue weight, body weight gain, food intake, liver lipid, and blood parameters. All values are means ± SE (*n* = 9). ^a^ *p* < 0.05, ^b^ *p* < 0.01, and ^c^ *p* < 0.001 compared with the low-fat diet (CON) group; ^d^ *p* < 0.05, ^e^ *p* < 0.01, and ^f^ *p* < 0.001 compared with the high-fat plus vehicle (distilled water) (HF; high-fat diet control) group. (−)-epicatechin-3-*O*-β-d-allopyranoside (BB): B1: 10, B2: 20, B3: 40 mg/kg body wt; Rosi: rosiglitazone (10 mg/kg body wt); Feno: fenofibrate (250 mg/kg body wt). BAT, brown adipose tissue; RWAT, retroperioneal white adipose tissue; MWAT, mesenteric white adipose tissue; FFA, plasm free fatty acid.

Parameter	CON	HF	HF + B1	HF + B2	HF + B3	HF + Rosi	HF + Feno
Dose (mg/kg/day)	0	0	10	20	40	10	250
**Absolute tissue weight (g)**							
EWAT	0.415 ± 0.029	1.303 ± 0.123 ^c^	0.906 ± 0.105 ^d^	0.902 ± 0.084 ^d^	0.936 ± 0.075 ^d^	0.919 ± 0.097 ^d^	0.763 ± 0.116 ^f^
MWAT	0.139 ± 0.024	0.310 ± 0.027 ^b^	0.293 ± 0.018	0.267 ± 0.021	0.236 ± 0.030	0.302 ± 0.029	0.264 ± 0.056
RWAT	0.089 ± 0.012	0.469 ± 0.057 ^c^	0.280 ± 0.043 ^d^	0.263 ± 0.034 ^f^	0.233 ± 0.026 ^f^	0.224 ± 0.032 ^f^	0.173 ± 0.032 ^f^
Visceral fat	0.504 ± 0.039	1.772 ± 0.173 ^c^	1.273 ± 0.147 ^d^	1.199 ± 0.104 ^e^	1.198 ± 0.075 ^e^	1.143 ± 0.124 ^e^	0.936 ± 0.147 ^f^
Skeletal muscle	0.662 ± 0.036	0.874 ± 0.078 ^a^	0.873 ± 0.060	0.813 ± 0.077	1.156 ± 0.102 ^d^	0.960 ± 0.078	0.990 ± 0.078
BAT	0.084 ± 0.008	0.171 ± 0.014 ^b^	0.168 ± 0.011	0.156 ± 0.017	0.144 ± 0.010	0.206 ± 0.012	0.139 ± 0.011
Liver (g)	0.879 ± 0.019	0.884 ± 0.035	0.899 ± 0.036	0.866 ± 0.036	0.848 ± 0.028	0.886 ± 0.031	1.699 ± 0.033 ^f^
Weight gain (g)	0.01 ± 0.19	0.89 ± 0.16 ^a^	0.40 ± 0.28	0.61 ± 0.43	−0.05 ± 0.11 ^d^	0.38 ± 0.32	−0.58 ± 0.48 ^d^
Body weight (g)	24.37 ± 0.46	28.80 ± 0.93 ^b^	27.85 ± 1.04	27.88 ± 1.14	27.15 ± 0.84	28.61 ± 1.51	26.92 ± 0.96
Food intake (g/day/mouse)	2.40 ± 0.04	2.14 ± 0.04 ^c^	2.08 ± 0.05	2.03 ± 0.03	2.02 ± 0.03	2.11 ± 0.06	2.14 ± 0.04
**Liver lipids**							
Total lipid (mg/g)	55.3 ± 4.5	99.1 ± 7.1 ^b^	71.9 ± 6.5 ^d^	66.1 ± 6.9 ^e^	60.2 ± 5.2 ^f^	70.1 ± 8.2 ^d^	62.4 ± 3.9 ^f^
Triacylglycerol (μmol/g)	42.1 ± 4.5	81.1 ± 7.8 ^b^	49.9 ± 8.0 ^d^	42.8 ± 6.5 ^f^	40.8 ± 4.6 ^f^	52.1 ± 7.7 ^d^	43.8 ± 5.6 ^f^
**Blood profiles**							
FFA (mEq/L)	1.02 ± 0.10	1.48 ± 0.23 ^b^	1.11 ± 0.13 ^d^	0.95 ± 0.04 ^f^	0.86 ± 0.09 ^f^	1.08 ± 0.11 ^e^	0.91 ± 0.08 ^f^
TC (mg/dL)	104.6 ± 8.6	163.8 ± 7.2 ^c^	153.5 ± 7.7	144.1 ± 4.0	142.9 ± 6.6	111.5 ± 2.3 ^f^	104.5 ± 5.5 ^f^
Leptin (ng/mL)	1.359 ± 0.044	2.133 ± 0.044 ^c^	1.662 ± 0.009 ^f^	1.372 ± 0.029 ^f^	1.321 ± 0.008 ^f^	1.661 ± 0.013 ^f^	1.206 ± 0.015 ^f^
Adiponectin (μg/mL)	2.737 ± 0.021	2.153 ± 0.036 ^c^	2.895 ± 0.010 ^f^	3.277 ± 0.052 ^f^	3.628 ± 0.034 ^f^	3.058 ± 0.040 ^f^	3.101 ± 0.028 ^f^

### 2.2. Glucose and Insulin Levels in Blood

After 12 weeks on HFD exposure, the HF group showed evidence of hyperglycemia and hyperinsulinenia. Treatment with B1, B2, B3, Rosi, and Feno markedly lowered the blood levels of glucose and insulin (Figure 2B,C).

### 2.3. Blood Lipid, Leptin, and Adiponectin Levels, and Hepatic Lipid

At 12 weeks of HFD exposure, the HF mice had enhanced circulating levels of triglycerides (TG) (Figure 2D), total cholesterol (TC), leptin, and free fatty acid, whereas there were decreased adiponectin levels (Table 1). Administration of BB, Rosi, and Feno decreased TG levels. Rosi- and Feno-treated mice had lower TC levels. The BB-, Rosi-, and Feno-administered mice displayed reduced blood concentrations of leptin, whereas they had enhanced blood levels of adiponectin. Feeding a HFD not only enhanced total lipids but also triacylglycerol levels in liver tissue. Administration of B1, B2, B3, Rosi, and Feno decreased total lipids and triacylglycerol in the liver (Table 1). 

**Figure 3:**
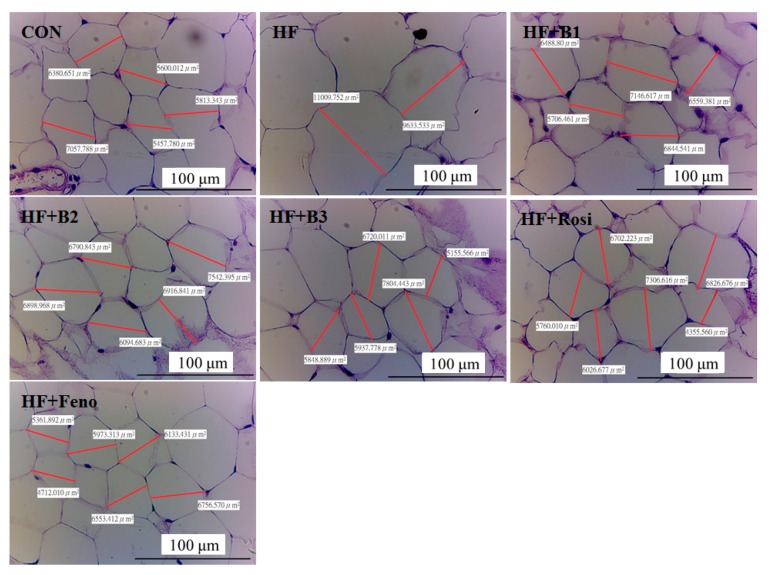
Pathological effects of (−)-epicatechin-3-*O*-β-d-allopyranoside (BB) on epididymal white adipose tissue (WAT) in the low-fat (CON), high-fat (HF; high-fat diet control), HF + B1, HF + B2, HF + B3, or HF + Rosi, and HF + Feno groups. Pictures of hematoxylin and eosin-stained sections of epididymal adipocytes (Magnification: 10 (ocular) ×20 (object lens)) from mice fed with BB. The appearance of adipocytes is polyhedral and displayed the string-like cytosol surrounded by a vacuole. Each presented is typical and representative of nine mice. (−)-Epicatechin-3-*O*-β-d-allopyranoside (BB): B1: 10, B2: 20, B3: 40 mg/kg body wt; Rosi: rosiglitazone (10 mg/kg body wt); Feno: fenofibrate (250 mg/kg body wt).

### 2.4. Pathological Investigation

After 12 weeks on HFD, HF mice caused the adipocytes to hypertrophy (the areas of HF mice and CON mice are 10216.4 ± 338.4 and 5347.5 ± 408.9 μm^2^, respectively), while mice administered with B1 (6882.8 ± 102.2 μm^2^), B2 (6475.5 ± 102.6 μm^2^), B3 (6192.7 ± 100.7 μm^2^), and Feno (5886.4 ± 58.7 μm^2^) displayed significant resistance to hypertrophy. The average data in Rosi-treated mice are 6982.3 ± 309.7 µm^2^ (Figure 3). Each image was carried out three times.

**Figure 4:**
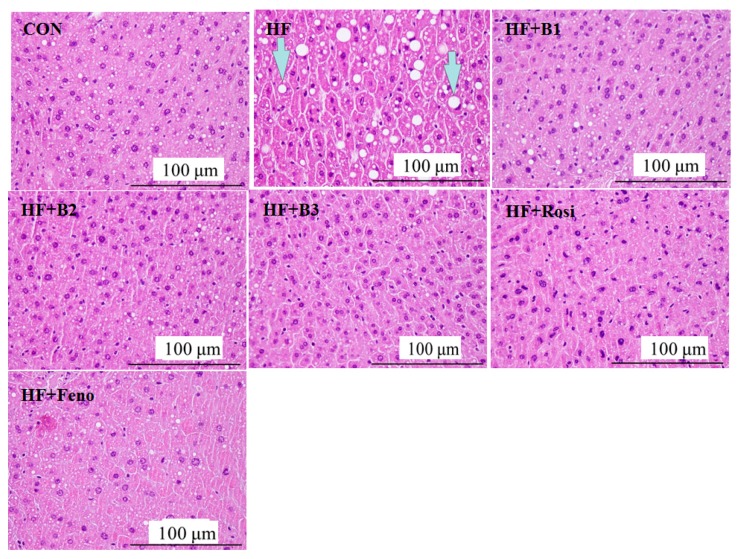
Pathological effects of (−)-epicatechin-3-*O*-β-d-allopyranoside (BB) on liver tissue morphology in the low-fat (CON), high-fat (HF; high-fat diet control), HF + B1, HF + B2, HF + B3, or HF + Rosi, and HF + Feno groups. Pictures of hematoxylin and eosin-stained sections of liver tissue (Magnification: 10 (ocular) × 20 (object lens)) from mice fed with (−)-epicatechin-3-*O*-β-d-allopyranoside (BB). The high-fat diet (HFD) induced obesity and insulin resistance. Each presented is typical and representative of nine mice. (−)-epicatechin-3-*O*-β-d-allopyranoside (BB): B1: 10, B2: 20, B3: 40 mg/kg body wt; Rosi: rosiglitazone (10 mg/kg body wt); Feno: fenofibrate (250 mg/kg body wt).

HFD caused significant ballooning degeneration of hepatocytes. Our findings show that HFD caused the ballooning degeneration in the liver of HF mice, which means hepatocyte death and accumulated glycogen in the center, and it was observed that the nucleolus was squeezed into the side, which is the so-called ballooning (the indicated arrow). Based on a previous report [28], the designation of histological hepatocellular ballooning findings included grade 0, none; grade 1, a few cells; grade 2, many cells. HFD caused ballooning evident in HF mice (mean score = 1.9 ± 0.2). The appearance of ballooning is lower in the B1-treated (0.7 ± 0.1), B2-treated (0.5 ± 0.2), B3-treated (0.4 ± 0.1), Rosi-treated (0.8 ± 0.2), and Feno-treated (0.6 ± 0.1) mice. Treatment with B1, B2, B3, Rosi, and Feno reduced the ballooning phenomenon (Figure 4).

### 2.5. Hepatic Related Gene mRNA Levels

**Figure 5:**
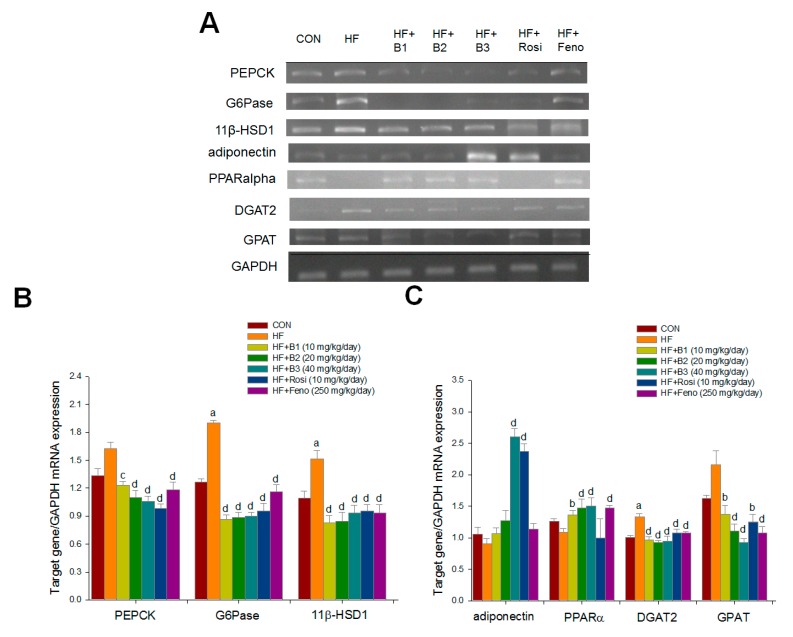
Semi-quantative transcription-polymerase chain reaction (RT-PCR) analysis on PEPCK, glucose-6-phosphatase (G6Pase), 11β-HSD1, adiponectin, PPARα, DGAT2, and GPAT mRNA expression in liver tissue of the mice receiving (−)-epicatechin-3-*O*-β-d-allopyranoside (BB) by oral gavage for four weeks. (**A**) Representative image; (**B**) and (**C**) Mean ± SE, target gene/GAPDH mRNA expression. All values are means ± SE (*n* = 9). ^a^ *p* < 0.001 compared with the low-fat diet (CON) group; ^b^ *p* < 0.05, ^c^ *p* < 0.01, and ^d^ *p* < 0.001 compared with the high-fat plus vehicle (HF; high-fat diet control) group. (−)-epicatechin-3-*O*-β-d-allopyranoside (BB): B1: 10, B2: 20, B3: 40 mg/kg body wt; Rosi: rosiglitazone (10 mg/kg body wt); Feno: fenofibrate (250 mg/kg body wt). Total RNA (1 μg) isolated from tissue was reverse transcripted by MMLV-RT, 10 μL of RT products were used as templates for PCR. Signals were quantitated by image analysis; each value was normalized by GAPDH.

HFD caused an increase in the mRNA levels of glucose-6-phosphatase (G6 Pase), and 11β hydroxysteroid dehydrogenase 1 (11β-HSD1). Administration of B1, B2, B3, Rosi, and Feno led to reduced mRNA levels of phosphenolpyruvate carboxykinase (PEPCK), G6 Pase, 11β-HSD1, acyl-coenzyme A: diacylglycerol acyltransferase (DGAT2), and glycerol-3-phosphate-acyltransferase (GPAT). B3 and Rosi treatments enhanced adiponectin mRNA levels. Administration of B1, B2, B3, and Feno enhanced PPARα mRNA levels (Figure 5A–C).

### 2.6. Western Blotting

**Figure 6:**
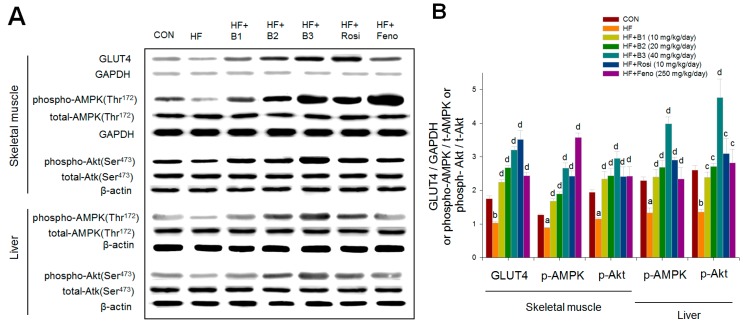
The protein contents of GLUT4 in skeletal muscle, and the expression levels of phospho-AMPK (Thr^172^)/total AMPK and phospho-Akt/total Akt in skeletal muscle and liver tissue of the mice receiving (−)-epicatechin-3-*O*-β-d-allopyranoside (BB) by oral gavage for four weeks. Protein was separated by 12% SDS-PAGE detected by Western blot. (**A**) Representative image; (**B**) Mean ± SE, GLUT4/GAPDH or phospho-AMPK/t-AMPK or Phospho-Akt/t-Akt expression levels. All values are means ± SE (*n* = 9). ^a^ *p* < 0.05, and ^b^ *p* < 0.001 compared with the low-fat diet (CON) group; ^c^ *p* < 0.05, and ^d^ *p* < 0.001 compared with the high-fat plus vehicle (HF; high-fat diet control) group. (−)-epicatechin-3-*O*-β-d-allopyranoside (BB): B1: 10, B2: 20, B3: 40 mg/kg body wt; Rosi: rosiglitazone (10 mg/kg body wt); Feno: fenofibrate (250 mg/kg body wt).

HFD caused a decrease in skeletal muscular expression levels of GLUT4. Following treatment with BB, Rosi, and Feno, membrane protein levels of GLUT4 were significantly enhanced. It was observed that HFD caused a decrease in expression levels of phospho-AMPK/total AMPK in both muscle and liver. Administration of BB, Rosi, and Feno enhanced expression levels of phospho-AMPK/total AMPK in these two tissues. The expression levels of phospho-Akt/total Akt in both muscle and liver tissue were lower in HF mice compared with CON mice. B1, B2, B3, Rosi, and Feno treatments displayed increases in both tissues in the expression levels of phospho-Akt/total Akt (Figure 6A,B).

## 3. Discussion

The aim of this study is to examine antidiabetic and antihyperlipidemic activity and molecular mechanisms of BB using high-fat diet (HFD)-fed mice. The present results indicate that HFD induced the hyperglycemia, hypertriglyceridemia, and hyperinsulinemia consistent with reported hyperglycemia, hyperlipidemia, hyperinsulinemia, hyperleptinemia, obesity, and excess circulating free fatty acid [29,30]. BB-treated HFD-fed mice had reduced blood insulin levels, concomitant with a decrease in blood glucose level, implying insulin hypersensitivity. Adiponectin has shown to enhance insulin sensitivity, and its decreased production is associated with insulin resistance [31]. Rosi has shown to be one of the thiazolinediones (TZDs) and acts as an insulin-sensitizing drug and increases blood concentrations of adiponectin in patients with type 2 diabetes [31]. In the present study, HFD elicited the hypoadiponectinemia, which was significantly increased by BB and Rosi. Collectively, these data further demonstrated that BB can provide a useful approach in enhancing insulin sensitivity. Moreover, BB treatment lowered circulating triglyceride levels, establishing that BB indeed displays antidiabetic and antihyperlipidemic activity.

Visceral obesity is demonstrated to act at the core of the metabolic syndrome’s pathogenesis including insulin resistance and hyperlipidemia, and it was observed that BB decreased fat accumulation with lowered EWAT weights as well as reduced visceral fat mass. Thus, BB is thought to have therapeutic potential to ameliorate visceral fat mass, insulin resistance, and dyslipidemia.

To examine the molecular mechanism of antidiabetic effects of BB, we investigated muscular expression levels of GLUT4. Since glucose uptake in skeletal muscle contributed to the main part of glucose disposal in peripheral tissues, skeletal muscular GLUT4 contents were monitored following BB treatment in HFD-fed mice, and were compared to Rosi, which is demonstrated to enhanced insulin sensitivity. GLUT4 is the major regulator of glucose levels in blood. The levels of GLUT4 assess the movement of the insulin-responsive GLUT4 to the plasma membrane [11]. In this study, HFD caused a decrease in muscular expression levels of GLUT4 in HFD-fed mice. We observed BB and Rosi treatments displayed 3.11-fold and 3.41-fold enhancement, respectively, in the protein contents of GLUT4, showing that increased protein contents of GLUT4 contribute to the effect of BB in increasing sensitivity to insulin and glucose uptake, thus leading to reductions in blood glucose levels.

On the other hand, Akt (PKB) has been shown to stimulate glucose uptake by influencing glucose transporter 4 (GLUT4) [32]. BB also caused enhanced expression levels of Akt phosphorylation in skeletal muscle, indicating that BB can stimulate glucose transport activity partly in the insulin-dependent manner.

Based on this, AMPK is demonstrated to be important in the metabolism of glucose and lipids, thus AMPK phosphorylation in both muscle and liver tissue by BB was studied. The antidiabetic drug metformin is used in the management of Type 2 diabetes mellitus (T2DM). Previous study demonstrated that metformin lowered glucose levels mainly by inhibiting liver glucose production and increased peripheral glucose uptake [16]. Metformin could enhance muscular activation of AMPK [16]. AMPK could induce GLUT4 redistribution to the cell membrane in an insulin-independent manner [16]. In this study, BB caused an increase in muscular expression levels of AMPK phosphorylation, presumably partly via promoting membrane translocation of GLUT4, and stimulating glucose uptake in an insulin-independent manner.

PEPCK and G6Pase are rate-limiting enzymes in the gluconeogenic process [33]. Renovation of enhanced PEPCK expression is thought to be a positive methodology in treating the diabetic state [34,35]. Metformin has been shown to activate AMPK, and this is followed by the decreased PEPCK and G6 Pase gene [36]. We also observed administration of BB-reduced mRNA levels of PEPCK and G6Pase, suggesting that BB-downregulated PEPCK and G6 Pase and inhibited hepatic glucose production could partly contribute to the effect of a decrease in blood glucose levels. Collectively, these data indicate that the lowered liver glucose production and the enhanced skeletal muscular glucose uptake offer a possible mechanism for the primary hypoglycemic effect of BB.

Moreover, the enzyme 11β-HSD1 transforms dehydrocorticosterone to corticosterone, and 11β-HSD1 knockout mice are reported to ameliorate insulin resistance [37]. We observed that BB-treated mice decreased the hepatic mRNA levels of 11β-HSD1, thus partly contributing to enhanced insulin sensitivity.

The hypolipidemic mechanisms of BB were determined through analysis of AMPK phosphorylation and targeted gene expression in liver tissue. Liver triglyceride content and blood free fatty acid were decreased in BB-treated mice. Metformin could cause hepatocyte-specific AMPK activation, and thus leads to the enhanced transcriptional activity of the PPARα on the expression and suppressed lipogenic enzyme expression [16]. DGAT2 is the endoplasmic membrane-bound enzyme responsible for catalyzing the terminal pace of triacylglycerol synthesis, and has a central role in intracellular accumulation [38,39]. DGAT2 potently stimulated TG synthesis, yielding an increase in intracellular TG, which accumulated in large cytosolic lipid droplets [40]. DGAT2 expression was stimulated by glucose and insulin [41]. DGAT2 gene expression increases during adipogenesis which is accompanied by an increase in TG synthesis [42]. Although direct evidence is lacking, it appears that DGAT2 is likely regulated by C/EBPα, a key transcriptional regulator of adipogenesis. Thus, the downregulation of DGAT2 seems to contribute to hepatic triglyceride output, resulting in decreasing blood TG levels. In this study, BB displayed increased hepatic AMPK phosphorylation. Moreover, BB protected mice from diet-induced increases in hepatic lipid contents, at least in part by increased mRNA levels of PPARα and a reduction in GPAT, which catalyzes the first step of glycerolipid synthesis and is a reasonably promising site for the influence of triacylglycerol (TAG) synthesis. As there are enhanced mRNA levels of GPAT in the liver, and alterations in the regulation of mitochondrial GPAT might lead to lipid metabolism disorder, including type 2 diabetes [43], we could not entirely decide whether mRNA inhibition of GPAT would confer that these similar results resemble GPAT inhibition. Nevertheless, BB treatment resulted in a decrease in hepatic GPAT mRNA levels, suggesting that BB is involved in GPAT and linked to TAG synthesis. These data indicating BB displayed hypolipidemic activity were possibly associated with the regulation of several hepatic genes' mRNA levels. Whether associated with fatty acid oxidation and lipogenesis, studies on the level of analysis by BB are now being undertaken.

It was found that blood levels of adiponectin were decreased in type 2 diabetes and obesity patients [44]. In addition to adiponectin-mediated enhanced glucose uptake and phosphorylation of AMPK [45], leptin-activated AMPK leads to enhanced fatty acid oxidation and reduced triacylglycerol accumulation [46]. In this study, BB treatment caused increases in blood adiponectin and decreases in blood leptin. Thus, a possible explanation is that BB-activated AMPK may be linked to leptin or adiponectin secretion (or mRNA) through AMPK activation.

The present results found that B3-treated mice had lower body weight gain. This may be due to a decrease in visceral fat mass. Nevertheless, B3 enhanced the weight of skeletal muscle and caused Akt activation. Akt activation has been shown to elicit skeletal muscle hypertrophy [47] and enhance liver fatty acid oxidation with decreased fat pads [48]. Previous study showed that Akt/mTOR-mediated skeletal muscle hypertrophy leading to increased insulin sensitivity[48] and a report with finding that mutation in the gene encoding Akt2 results in serve insulin resistance [49], establishing that BB elevated Akt activation leads to muscular hypertrophy and enhanced insulin sensitivity, opposite to weight gain.

Pathology examinations have revealed that BB treatment displayed decreased adipocyte hypertrophy, and reduced hepatic balloon degeneration. It is known that the liver is the main tissue to metabolize fat and cause the blood TG level to fluctuate. A possible explanation is that BB promoted fat movement from visceral adipose to the liver via enhanced lipid metabolism in the liver with less steatosis while decreasing the areas of adipocytes.

In summary, the present study demonstrated that administration of BB could enhance expression levels of phospho-AMPK/total-AMPK in muscle and liver and membrane protein levels of GLUT4 in diabetic and hyperlipidemic mice. The present results demonstrated that BB could be an important intervention in the alleviation of diabetes and hyperlipidemia.

## 4. Experimental Section

### 4.1. Preparation of (−)-Epicatechin-3-O-β-d-allopyranoside (BB)

#### 4.1.1. Preparation of Extract of *Davallia formosana*

The pure compound of BB employed in the animal models was provided by Jin-Bin Wu. The roots and stems of *Davallia formosana* were obtained from a local market in Taichung, Taiwan; after identification, they were extracted with 75% ethanol and reduced pressure. The yield of the extract of *D. formosana* (DFE) was 9.5 wt % [50]. The ethanol extract was suspended in water and partitioned by *N*-butanol and concentrated; the yield of *N*-butanol fraction obtained from the ethanol extract is 20.2 wt % [50].

**Table 2:** The ^13^C- and ^1^H-NMR spectrum of (−)-epicatechin-3-*O*-β-d-allopyranoside (BB).

C		H	
C-2	79.19	H-2	5.05 (d, *J* = 2.2 Hz)
C-3	73.40	H-3	4.43 (m)
C-4	24.75	H-4-2	2.74, 2.72
C-5	157.85	-	-
C-6	96.43	H-6	5.86 (d, *J* = 2.3 Hz)
C-7	157.85	-	-
C-8	95.68	H-8	5.90 (d, *J* = 2.3 Hz)
C-9	157.13	-	-
C-10	100.21	-	-
C-1ʹ	131.67	-	-
C-2ʹ	115.57	H-2ʹ	7.02 (d, *J* = 2.1 Hz)
C-3ʹ	145.50	-	-
C-4ʹ	145.72	-	-
C-5ʹ	116.29	H-5ʹ	6.66 (d, *J* = 8.2 Hz)
C-6ʹ	120.34	H-6ʹ	6.78 (dd, *J* = 2.1 Hz)
Allosyl			
C-1ʹʹ	100.41	H-11ʹʹ	-
C-2ʹʹ	72.28	(H-2ʹʹ-6ʹʹ)	-
C-3ʹʹ	72.91	(H-5)	
C-4ʹʹ	68.96		
C-5ʹʹ	75.33		4.74 (d, *J* = 8 Hz)
C-6ʹʹ	63.26		3.22–3.99

#### 4.1.2. Purification of (−)-Epicatechin-3-*O*-β-d-allopyranoside

The *N*-butanol fraction (10 g) was introduced into an HP-20 column (Diaion, NIPPON RESSUI Company, Tokyo, Japan) and eluted with water, and followed by methanol [50]. Eight fractions were obtained (Fractions 1–8). Fraction 6 (230 mg) was purified by a preparative high performance liquid chromatograph (HPLC) (Shimadzu CL-8A, Kyoto, Japan) to obtain pure compounds (136 mg) [50]. The conditions of the preparative HPLC were as previously described [50].

The pure compound was analyzed by NMR (^1^H, ^13^C; Bruker ADVANCE DPX-200, Rheinstetten, Germany), and it was identified as (−)-epicatechin-3-*O*-β-d-allopyranoside (BB) (Figure 1) [50]. The result of the ^13^C- and ^1^H-NMR spectrum (200 MHz, CDCl_3_) of BB is shown in Table 2 and some modifications from a previous described [50]. Previous studies had shown that DFE contained an amount of BB, which was the active compound of DFE; nevertheless, naringin was not found in DFE [7,50,51].

### 4.2. Animal and Diet Treatment

This experiment was performed and approved by the guidelines of the Institutional Animal Care and Use Committee of Central Taiwan University of Science and Technology (13 May 2014) as previously described [52,53,54,55,56,57]. Male C57BL/6J mice (total amount = 63), at the age of four weeks, were purchased from the National Laboratory Animal Breeding and Research Center (Taipei, Taiwan) [52]. After acclimatization for one week, all of the mice were divided randomly into the control (CON) group (low-fat diet (control diet, CD)) and the high-fat diet (HFD) group [52]. The CON group (*n* = 9) was kept on a low-fat diet (Diet 12450B, Research Diets, Inc., New Brunswick, NJ, USA), while the HFD group (*n* = 54, including six groups) was exposed to a 45% high-fat diet (Diet 12451, Research Diets, New Brunswick, NJ, USA) for 12 weeks [52,56,57]. The control diet contained 10% fat, whereas HFD contained 45% fat (of total energy, % kcal); and the diet compositions and energy percentages were as previously described [52,53,56,57]. After HFD exposure for eight weeks, the HFD-fed group was further randomly divided into six groups (*n* = 9 per group), including dosing of BB (including B1: 10, B2: 20, and B3: 40 mg/kg/day body wt) or rosiglitazone (Rosi; 1% methylcellulose 10 mg/kg body wt, from GlaxoSmithKline) or fenofibrate (Feno; 250 mg/kg/day body wt, from Sigma Chemical Co, St. Louis, MI, USA) or vehicle (equal volumes of water) by oral gavage once daily for four weeks and on HFD [52]. The CON and high-fat control (HF) mice were merely administered vehicle [52]. After dosing for four weeks, we removed food from the mice at night; and on the next day, the mice after 12 h of fasting were sacrificed. All of the individual tissues were collected and weighed, and portions were instantly frozen at −80 °C for later target gene analysis [52]. A portion of the acquired blood samples (0.8 mL) were immediately taken for glucose level analysis, and a portion for TG, TC, insulin, leptin, and adiponectin concentration analysis [52].

### 4.3. Measurement of Body Weight, Body Weight Gain, and Diet Consumption

Body weight, body weight gain, and diet consumption were measured as previously described [52,53,54,55,56,57].

### 4.4. Analysis of Blood Glucose, Blood Lipid, Insulin, Leptin, and Adiponectin

A portion of obtained blood samples from the retro-orbital sinus of fasting mice were used to measure blood glucose levels as previously described [52,56,57]. Plasma TG, total cholesterol (TC), and free fatty acids were determined as previously described [52,56,57]. Plasma insulin, leptin, and adiponectin levels were assessed using an assay kit (mouse insulin ELISA kit, Mercodia, Uppsala, Sweden; mouse leptin ELISA kit, Morinaga, Yokohama, Japan; Mouse Adiponectin ELISA kit, Crystal Chem International, Downers Grove, IL, USA) as previously described [52,56,57].

### 4.5. Histology

Portions of EWAT and liver tissue specimens were monitored as previously described [50,52,57]. Microscopic images were taken using a microscope (Olympus BX51, BX51, Olympus, Tokyo, Japan).

### 4.6. Hepatic Lipids Analysis

This part of the analysis is presented in previous studies [52,54,56,57].

### 4.7. Relative Quantization of mRNA Analysis

Firstly, the total RNA from the liver tissue was isolated with a Trizol reagent (Molecular Research Center, Inc., Cincinnati, OH, USA), and this experiment was determined as described previously [52,53,54,55,56,57,58]. The primers are shown in Table 3.

**Table 3:** Primers used in this study.

Gene	Accession Number	Forward Primer and Reverse Primer	PCR Product (bp)	Annealing Temperature (°C)
Liver				
*PEPCK*	NM_011044.2	F: CTACAACTTCGGCAAATACC	330	52
		R: TCCAGATACCTGTCGATCTC		
*G6Pase*	NM_008061.3	F: GAACAACTAAAGCCTCTGAAAC	350	50
		R: TTGCTCGATACATAAAACACTC		
*11β-HSD1*	NM_008288.2	F: AAGCAGAGCAATGGCAGCAT	300	50
		R: GAGCAATCATAGGCTGGGTCA		
*Adiponectin*	NM_009605.4	F: TCTTCTACAACCAACAGAATCA	324	50.5
		R: GTATCATGGTAGAGAAGGAAGC		
*PPARα*	NM_011144	F: CCTGAGATTAACCAGCCTTT	352	55
		R: AGGACCTACTCTCATTGCTG		
*GPAT*	BC019201.1	F: CAGTCCTGAATAAGAGGT	441	48
		R: TGGACAAAGATGGCAGCAGA		
*GAPDH*	NM_008084.3	F: TGTGTCCGTCGTGGATCTGA	99	55
		R: CCTGCTTCACCACCTTCTTGA		

### 4.8. Western Blotting Analysis

This experiment was analyzed as previously described [52,53,54,55,56,57,58]. Finally, antibodies were detected using alkaline phosphatase linked to a goat anti-IgG rabbit secondary antibody [52,56,57] and detected by 5-bromo-4-chloro-3-indolyl-phosphate/ nitro blue tetrazolium (BCIP/NBT).

### 4.9. Statistical Analysis

All results were presented as the mean and standard error. Moreover, analysis of variance for data was employed and followed by Dunnett’s multiple range tests using SPSS software (SPSS Inc., Chicago, IL, USA). *p* < 0.05 is recognized as statistically significant.

## 5. Conclusions

Our findings revealed for the first time that BB lowered blood levels of glucose and insulin, concomitant with decreased blood triglyceride and lipid profiles, and ameliorated insulin resistance and dyslipidemia in mice on HFD. The skeletal muscular membrane expression level of GLUT4 was reduced in the HFD-fed group, which was reversed by BB treatment and correction of hyperglycemia. BB not only increased expression levels of phospho-AMPK/total-AMPK in skeletal muscle, but also in the liver. In addition to an increase in skeletal muscular protein levels of GLUT4 to enhance glucose uptake, a reduction in mRNA levels of PEPCK and the inhibition of hepatic glucose production leads to lowering blood glucose levels. Moreover, BB reduced the mRNA expressions of DGAT2 and GPAT (glycerolipid synthesis) while enhancing the mRNA expressions of PPARα (fatty acid oxidation) in liver tissue, which may be associated with decreasing blood TG levels. Further study will be undertaken on these levels of analysis. Nevertheless, our findings provide new insights, besides the prevention of osteoporosis, into understanding that BB could control and prevent type 2 diabetes and hyperlipidemia and hepatic fat accumulation.
